# Do Cognitive Abilities Influence Physical and Mental Fatigue in Patients with Chronic Pain after Walking According to a Clinical Guideline for Physical Exercise?

**DOI:** 10.3390/ijerph182413148

**Published:** 2021-12-13

**Authors:** Patricia Catala, Lorena Gutierrez, Carmen Écija, Ángel Serrano del Moral, Cecilia Peñacoba

**Affiliations:** 1 Department of Psychology, Universidad Rey Juan Carlos, Avda. de Atenas s/n, 28922 Madrid, Spain; patricia.catala@urjc.es (P.C.); lorena.gutierrezh@urjc.es (L.G.); carmen.ecija@urjc.es (C.É.); 2 General Surgery and Digestive Surgery Service, Hospital Universitario de Fuenlabrada, 28944 Madrid, Spain; aserranom@salud.madrid.org

**Keywords:** physical fatigue, mental fatigue, cognitive fusion, pain acceptance, walking, fibromyalgia, chronic pain

## Abstract

The objective of this study is to explore the mediator role of cognitive fusion and chronic pain acceptance on the effects that the walking pattern, following an established clinical guideline for physical exercise, can have on fatigue (physical and mental) in patients with chronic pain. The sample consisted of a total of 231 women with fibromyalgia with a mean age of 56.91 years (Standard Deviation SD = 9.58 years, range 30−78 years). The results show a significant indirect effect of the walking pattern on both physical and mental fatigue through cognitive fusion and chronic pain acceptance. Specifically, walking predicted less cognitive fusion, which predicted greater chronic pain acceptance, which, in turn, predicted less mental and physical fatigue (Beta-B- = −0.04, Standard Error SE = 0.02, 95% Confidence Interval 95% CI = [−0.09, −0.02]; B = −0.09, SE = 0.05, 95% CI = [−0.22, −0,15], respectively). It can be concluded that the walking pattern is linked to both physical and mental fatigue through cognitive defusion and chronic pain acceptance. These cognitive abilities would allow fibromyalgia patients to perceive an improvement in both physical and mental fatigue by carrying out the walking pattern. Emphasizing the training of cognitive defusion and pain acceptance would improve the adherence of these patients to walking.

## 1. Introduction

Physical therapy is chosen by health professionals as one of the main interventions to mitigate some of the symptoms associated with fibromyalgia (FM) [1,2]. Health professionals endorsed by the World Health Organization (WHO) frequently recommend walking for 30 minutes or more at least five days a week [3] due to its low musculoskeletal impact and the benefits it has on pain management, mood or fatigue [4]. Specifically, in patients with fibromyalgia, it is recommended that they walk at least 30 min twice a week, for a minimum of six consecutive weeks. In addition, it is suggested that to avoid fatigue they establish small breaks. Despite all these benefits being known, studies show that women with fibromyalgia do not always perceive an improvement in their physical and/or mental health after doing physical exercise [5,6] and point out this reason as one of the main inhibitors of walking [7,8,9].

Recently, a model of psychological flexibility has been proposed as a unifying framework that could help understand this question. Psychological flexibility refers to the ability of an individual to consciously stay in touch with the present moment [10]. This ability is developed from a set of skills that allow us to accurately predict how other mental or behavioral processes will develop. Cognitive fusion and acceptance are two of the cognitive skills that have generated increasing interest in the literature. The first refers to assuming as true all the thoughts that arise in the mind and acting according to them [11,12,13]. That is, we believe that we are what we think or that what we think is literally reality. The second can be defined as the ability to be with the experience (emotions, thoughts and physical sensations), while the efforts to control or avoid it cease [14]. Thus, it is to let it be, to give it its space, to observe it without judgment, understanding that it appears as an answer.

Among the studies carried out in patients with chronic pain, it has been found that cognitive psychological processes play an important role in the daily functioning of women with fibromyalgia and in how they relate to their disease [15]. Specifically, cognitive fusion has been negatively related to a commitment to activities [16,17], fatigue [15], well-being [16] and general health perceived as worse [18]. The acceptance of chronic pain has been related to an improvement in the quality of life related to physical and mental health [19,20] and to the pain-related functioning and disease impact [21,22]. Likewise, some studies mention that both variables could mediate the perception of health improvements [21,23]. In particular, this influence of cognitive variables has been specially studied in relation to pain as it constitutes the most characteristic symptom of the fibromyalgia diagnosis. In opposition to pain, fatigue has been the focus of just a small number of studies [24], although this symptom has been shown to be highly prevalent, persistent and disabling in patients with fibromyalgia [25,26,27]. Therefore, the objective of this study was to analyze the mediating effect of personal skills, specifically, cognitive fusion and acceptance of pain, between the recommended pattern of walking and both physical and mental fatigue in patients with fibromyalgia. As hypotheses, it is proposed that both variables mediate the relationship between carrying out the walking pattern and physical and mental fatigue.

## 2. Materials and Methods

### 2.1. Participants

The sample consisted of 231 women with FM with a mean age of 56.91 years (SD = 9.58 years, range 30–78 years). Eligibility criteria to participate in the present study included having a diagnosis of FM according to the criteria of the American College of Rheumatology (ACR) [28,29], having medical advice for walking, not having a physical comorbidity or any other pathology that prevents carrying out the walking pattern, being over 18 years of age, and providing written consent to participate in the research. Patients were recruited from different mutual aid associations in Spain (Madrid, Ciudad Real, Albacete, Guadalajara and Toledo). Most women (79%) were married or in a stable relationship, 15% were divorced or widows, and 6% were single. Regarding the level of studies, six percent of the women had completed university studies, 26.6% had a secondary school education, 51.3% had a primary education, and 15.6% knew how to read and write. Participants had a diagnosis of fibromyalgia for an average of 13.02 years (SD = 7.21, range 1 to 46 years).

### 2.2. Measures

#### 2.2.1. Walking Pattern

An ad hoc question was used. Taking into account the fact that this population is sedentary, the guideline established by Gusi [4] for patients with fibromyalgia was established as a criterion: “walking at least 30 min, twice a week, over a minimum of six consecutive weeks. To make it easier to adhere to this minimum guideline, it is convenient that you establish small pauses to prevent fatigue”. They were asked to answer yes or no based on having followed this pattern in recent months.

#### 2.2.2. Chronic Pain Acceptance Questionnaire (CPAQ)

The total score of the Chronic Pain Acceptance Questionnaire (CPAQ) was used [30]. This instrument consists of 20 items that are valued on a Likert scale from 0 (it is never true) to 6 (it is always the case), obtaining three scores: one total, and another two for the subscales of involvement in activities (IA) and opening to pain (AD). This is a self-report assessment used to assess pain acceptance in people with chronic pain (i.e., “My life is fine even though I have chronic pain”, “Although things have changed, I am living a normal life despite my chronic pain”). Higher scores indicate a greater acceptance of pain (the total scores on the scale range from 0 to 54). In the present study, Cronbach’s alpha was 0.87.

#### 2.2.3. Cognitive Fusion

The Spanish version of the Cognitive Fusion Questionnaire was used [12,31]. This questionnaire contains 7 items rated on a 7-point Likert scale that are rated from 1 (never) to 7 (always). The theoretical range of the instrument is between 7 and 49, and high total scores indicate a high cognitive fusion. The scale contains items such as “I feel so caught up in my thoughts that I can’t do the things I want to do the most” or “I struggle with my thoughts”. In the current study, the Cronbach’s alpha was 0.88.

#### 2.2.4. Mental and Physical Fatigue

The Physical Fatigue and Mental Fatigue dimensions of the Spanish version of the Multidimensional Fatigue Inventory (MFI) were used [32]. This questionnaire is a 20-item assessment tool with a 5-point Likert response format ranging from 1 = “Yes, that is true” to 5 = “No, that is not true”, and evaluates five factors. The factors are: general fatigue, physical fatigue, mental fatigue, reduced motivation and reduced activity. Each factor is made up of four items, so the range for each of them oscillates between 5 and 20. High scores on the dimension indicate a high degree of fatigue symptoms. For this study, the factors of physical fatigue containing elements such as “Physically I feel only able to do a little” and mental fatigue containing elements such as “It takes a lot of effort to concentrate on things” were selected. The selection of these dimensions was motivated by their conceptualization of fatigue as a physical symptom on the one hand, and as a cognitive symptom on the other hand. Mental and physical fatigue are consistent with the theoretical model of fatigue in fibromyalgia and, specifically, with the previous literature on the relationship between (1) fatigue and walking behavior [4] and (2) fatigue and cognitive processes [15,19,20]. In the present work, the internal consistency for both subscales was good (0.83 for mental fatigue and 0.80 for physical fatigue).

#### 2.2.5. Socio-Demographic and Clinical Data

An ad-hoc questionnaire was used to evaluate the age, marital status, educational level and employment status. Regarding clinical variables, the duration of fibromyalgia was recorded.

### 2.3. Procedure

A total of 685 members from eleven Spanish fibromyalgia associations, which collectively comprise 1471 members with a clinical diagnosis of fibromyalgia (a compulsory requisite to join the association), satisfied the inclusion criteria for this study. Recruitment was made via mail and phone. Out of the 685 eligible participants, we were unable to contact twelve and 122 refused to participate. Thus, our population comprised of 551 women with fibromyalgia who were all contacted by ordinary mail, email and phone through the associations. Finally, 231 attended the appointment (41.92%).

Researchers traveled to the associations and cited patients in groups of 10–12 patients (3 researchers per group). The patients received a protocol with the questionnaires that measured the variables of interest, and the researcher was present in case there was any difficulty in completing it. Finally, the researcher reviewed the protocols once they were completed to ensure that there was no missing data. Completion of the protocols lasted between 30 and 45 minutes.

### 2.4. Statistical Analysis

The analyses were performed with the SPSS 22 statistical package [33]. First, a descriptive analysis, internal consistency analysis (Cronbach’s alpha coefficients) and Pearson correlations were performed. For continuous variables, means, standard deviations and range medians were used, while categorical data were presented as numbers and percentages. The level of statistical significance for all tests was established at a *p* value of less than 0.05.

For the serial multiple mediation analysis (SMM; mediation of several mediators going one after another), SPSS macro PROCESS (model 6) was used, applying two significant mediators. As recommended by Hayes [34], the regression/trajectory coefficients are all in nonstandardized form since the standardized coefficients generally do not have a useful substantive interpretation. The tested model included “walk regularly for at least 30 minutes twice a week, over a minimum of six consecutive weeks” as a predictor (X), cognitive fusion (M1) and chronic pain acceptance (M2) as mediators, and physical and mental fatigue as dependent variables (Y and Y‘, respectively). The model fit was also examined using the following criteria: a chi-square/df of <= 2, a *p*-value of > 0.05, a comparative fit index of > = 0.95, and an approximation of the mean squared error of <0.06 [35].

## 3. Results

### 3.1. Descriptive Analysis and Correlations of the Variables under Study

Table 1 shows the mean values, SD and ranges of the psychosocial variables. 

There was a significant correlation between the scores of all the variables under study. Walking was negatively correlated with cognitive fusion (*r* = −0.177, *p* = 0.008), as well as with mental (*r* = −0.41, *p* < 0.001) and physical (*r* = −2.67, *p* < 0.001) fatigue, and was positively correlated with chronic pain acceptance (*r* = 0.27, *p* < 0.001). Both mental and physical fatigue were positively correlated with cognitive fusion (*r* = 0.26, *p* < 0.001 and *r* = 0.427, *p* < 0.001, respectively) and negatively with chronic pain acceptance (*r* = −0.32, *p* < 0.001 and *r* = −0.28, *p* < 0.001, respectively). Regarding the sociodemographic variables (age, marital status, educational level), no significant differences were observed regarding the variables under study (all *p* > 0.05).

Following the recommendations of Baron & Kenny [36] for the inclusion of mediator variables, there must be a significant correlation with both predictors and outcome variables. Therefore, the two mediators proposed here were analyzed with predictive variables (walking) and criteria (physical and mental fatigue), in order to assess whether they should be included in the model. As the analyses described above show, both cognitive fusion and chronic pain acceptance could be included in the path model; therefore, they were included for the mediation analysis.

### 3.2. SMM Analysis

**Figure 1:**
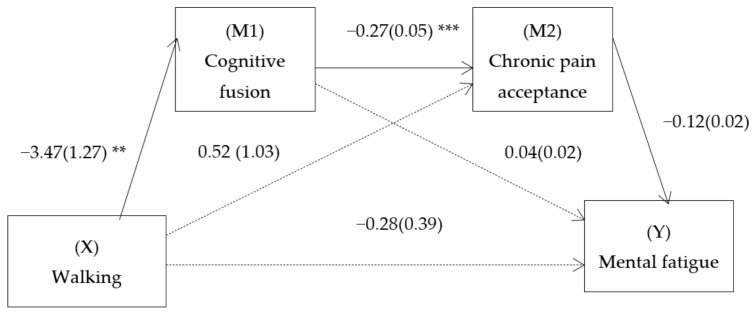
Path diagram illustrating the direct effects and causal paths linking walking (for at least 30 minutes twice a week, over a minimum of six consecutive weeks) with mental fatigue. Notes: Serial multiple mediation analysis with walking as the independent variable, mental fatigue as the dependent variable, and cognitive fusion and acceptance as the first and second mediators. Values are unstandardized regression coefficients (SE in parentheses) and associated *p* values (** *p* < 0.01, *** *p* < 0.001). Bracketed association=direct effect (controlling for indirect effects). Solid lines indicate significant pathways, and dashed lines indicate nonsignificant pathways.

Given that two mediators were used and taking into account the theoretical basis explained above to establish the causal order of the mediators, two models were proposed: SMM 1 (walking—cognitive fusion—chronic pain acceptance—mental fatigue) and SMM 2 (walking—cognitive fusion—chronic pain acceptance—physical fatigue). For the SMM1 model, a total effect (B = −1.51, SE = 0.41, *t* = −3.12, 95% CI = [−2.41, −0.86], *p* = 0.003) of the predictors on mental fatigue was observed. The effect of walking on mental fatigue was completely mediated by the two serial mediators (B = −0.04, SE = 0.02, 95% CI = [−0.09, −0.02]) (Figure 1). That is, walking predicted less cognitive fusion, which predicted greater chronic pain acceptance, which, in turn, predicted less mental fatigue. No simple indirect effect was observed through any independent mediator. There was also no direct effect of walking on mental fatigue.

**Figure 2:**
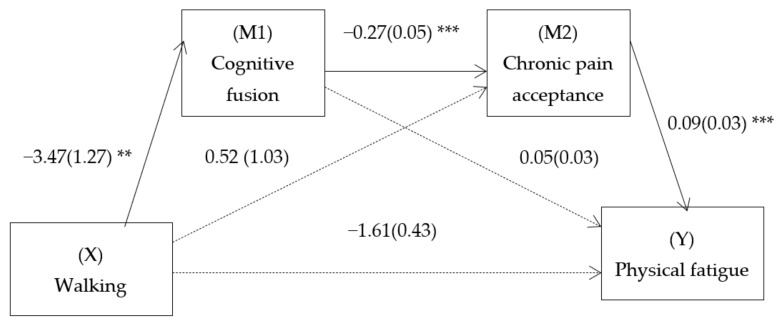
Path diagram illustrating the direct effects and causal paths linking walking (for at least 30 minutes twice a week, over a minimum of six consecutive weeks) with physical fatigue. Notes: Serial multiple mediation analysis with walking as the independent variable, physical fatigue as the dependent variable, and cognitive fusion and acceptance as the first and second mediators. Values are unstandardized regression coefficients (SE in parentheses) and associated *p* values (** *p* < 0.01, *** *p* < 0.001). Bracketed association=direct effect (controlling for indirect effects). Solid lines indicate significant pathways, and dashed lines indicate nonsignificant pathways.

For the SMM 2 model, a direct effect of walking on physical fatigue was found (B = −1.61, SE = 0.43, *t* = −3.73, 95% CI = [−2.45, −0.76], *p* = 0.003) and a total effect (B = −1.84, SE = 0.44, *t* = −4.13, 95% CI = [−2.71, −0.96], *p* < 0.001) of the predictors was found on physical fatigue. Furthermore, there was a significant indirect effect of walking on physical fatigue through cognitive fusion and chronic pain acceptance (B = −0.09, SE = 0.05, 95% CI = [−0.22, 0.15]) (Figure 2). That is, walking predicted less cognitive fusion, which predicted greater acceptance, which, in turn, predicted less physical fatigue. No simple indirect effect was observed through any independent mediator.

## 4. Discussion

The main objective of this study was to analyze the relationships established between walking (following an established clinical guideline for physical exercise) [4], cognitive fusion, pain acceptance, and physical and mental fatigue in patients with fibromyalgia. The results obtained in accordance with the proposed hypothesis show that there is a significant indirect effect of walking on the two fatigue components, mediated by cognitive fusion and pain acceptance. That is, carrying out the walking pattern recommended by health professionals predicted lower scores in cognitive fusion. This predicted greater acceptance of pain, which, in turn, predicted lower levels of physical and mental fatigue. Therefore, it could be said that women with fibromyalgia who have the ability to reduce those unwanted thoughts that appear in their minds and to be focused on the experience without trying to control or avoid pain, are capable of perceiving an improvement in their state of health, both physical and mental, after performing structured physical exercise (i.e., walking). This finding supports the model of psychological flexibility that, as mentioned above, emphasizes the importance of developing cognitive skills to accurately explain how other mental or behavioral processes will develop [10]. This has important clinical repercussions, since it corroborates the importance of carrying out multidisciplinary biopsychosocial treatments in the population with chronic pain [37,38,39,40]. Thus, combining pharmacological treatment with structured physical exercise therapy and acceptance and commitment therapy (ACT) could be a more beneficial alternative for improving the quality of life of patients in relation to physical and mental health. In this specific case, the ACT approach is recommended as it encompasses methods based on the psychological flexibility model from a functional contextual perspective of behavior (i.e., cognitive defusion techniques or mindfulness exercises). From this approach, a new way of responding to the world is trained by adopting an open, focused and committed response style [41,42]. The most recent results have already demonstrated the efficacy of these techniques in patients with chronic pain [43,44].

Another of the obtained findings shows that there were no simple indirect effects of walking on the fatigue components through any mediator acting independently. There was also no direct effect of walking on mental fatigue. Although there are a number of professionals who recommend physical therapy as a strategy to alleviate mental fatigue [45], the results obtained here indicate that in patients with fibromyalgia, walking without having certain cognitive abilities has no effect on mental fatigue. This could mean that for these women to be able to perceive walking as beneficial for mental clearing, they need to possess various cognitive processing skills (i.e., cognitive defusion and acceptance of chronic pain), and that it is not enough to manage a single cognitive ability or none at all.

An interesting piece of information from this study is the differences between the direct effects of walking on physical and mental fatigue. While the results do not show a significant direct effect of walking on mental fatigue, a significant direct effect of walking was found on the physical fatigue component. As established in the previous literature, the results indicate that the performance of the aforementioned gait pattern with the aim of performing physical exercise reduces the perception of self-reported fatigue [46]. On the other hand, it is also well known that one of the main reasons why patients do not adhere to the gait pattern is due to the lack of a perception of improvements related to their health after carrying out the activity [5,6,7,8,9]. The lack of continuity in the walk could explain this fact. As already hypothesized by Terrier [47], unstructured walking practiced in many discontinuous episodes may not have the same positive effects as structured walking exercises of longer duration. Research is needed to clarify this question.

While it is true that this study has important practical implications, a number of limitations must be taken into account. First, the results are based on women with FM, and though they constitute the most common sex for this condition, more research in other populations with chronic pain and in men is considered necessary to see if the found results are generalizable. Second, this is a cross-sectional design, so it does not allow one to infer cause–effect relationships between walking pattern, fatigue, cognitive fusion and acceptance of pain. Longitudinal studies are encouraged in order to investigate the causal relationships. Third, the assessment instruments are self-report measures, so they are subject to response bias. In future research, it would be interesting, especially in relation to the pattern of walking, to carry out longitudinal studies, completing the information with accelerometers/pedometers. Finally, it is important to note that the study included two mediators, defusion and chronic pain acceptance, in relation to the cognitive abilities of the patients, but the list is far from complete, and other psychosocial factors (i.e., motivation, beliefs, coping or psychological distress, and fear of movement) could also be investigated in future research.

Taking into account the above limitations and paying special attention to those related to the study design, it could be hypothesized that the performance of structured physical activity could predict improvements in physical and mental fatigue through cognitive defusion and chronic pain acceptance. Therefore, it is recommended that one carry out multicomponent interventions that integrate structural physical therapy and therapies framed within the model of psychological flexibility. This could also have a positive influence on patients, who could interpret physical exercise as beneficial for their physical and mental health and thus improve their adherence to walking.

## Figures and Tables

**Table 1:** Descriptive statistics for psychosocial characteristics (*n* = 231).

Psychosocial Characteristics	
Walking, n (%)	
Yes	132 (57.1)
No	95 (41.1)
Cognitive fusion, mean (SD)	33.31 (9.61)
Chronic Pain Acceptance, mean (SD)	18.93 (7.84)
Mental fatigue, mean (SD)	15.25 (3.15)
Physical fatigue, mean (SD)	15.21 (3.39)

SD: Standard deviation.

## Data Availability

The data presented in this study are available on request from the corresponding author. The data are not publicly available due to privacy restrictions.
